# Neuroprotective Effects of Black Pepper and Its Bioactive Compounds in Age-Related Neurological Disorders

**DOI:** 10.14336/AD.2022.1022

**Published:** 2023-06-01

**Authors:** Rengasamy Balakrishnan, Shofiul Azam, In-Su Kim, Dong-Kug Choi

**Affiliations:** ^1^Department of Applied Life Science, Graduate School, BK21 Program, Konkuk University, Chungju 27478, Korea.; ^2^Department of Biotechnology, College of Biomedical and Health Science, Research Institute of Inflammatory Disease (RID), Konkuk University, Chungju 27478, Korea.

**Keywords:** black pepper, bioactive constituents, neuroprotective, therapeutic, age-relate neurological disorders

## Abstract

Age-related neurological disorders (ANDs), including neurodegenerative diseases, are multifactorial disorders whose risk increases with age. The main pathological hallmarks of ANDs include behavioral changes, excessive oxidative stress, progressive functional declines, impaired mitochondrial function, protein misfolding, neuroinflammation, and neuronal cell death. Recently, efforts have been made to overcome ANDs because of their increased age-dependent prevalence. Black pepper, the fruit of *Piper nigrum L*. in the family Piperaceae, is an important food spice that has long been used in traditional medicine to treat various human diseases. Consumption of black pepper and black pepper-enriched products is associated with numerous health benefits due to its antioxidant, antidiabetic, anti-obesity, antihypertensive, anti-inflammatory, anticancer, hepatoprotective, and neuroprotective properties. This review shows that black pepper’s major bioactive neuroprotective compounds, such as piperine, effectively prevent AND symptoms and pathological conditions by modulating cell survival signaling and death. Relevant molecular mechanisms are also discussed. In addition, we highlight how recently developed novel nanodelivery systems are vital for improving the efficacy, solubility, bioavailability, and neuroprotective properties of black pepper (and thus piperine) in different experimental AND models, including clinical trials. This extensive review shows that black pepper and its active ingredients have therapeutic potential for ANDs.

## 1. Introduction

Neurological disorders affect almost one billion people worldwide, and aging is a major risk factor [1]. Age-related neurological disorders (ANDs) include neurodegenerative diseases (NDDs) such as Alzheimer’s disease (AD), Parkinson’s disease (PD), Huntington’s disease (HD), and multiple sclerosis (MS). ANDs, such as epilepsy and ischemic stroke, and neuropsychological disorders, such as depression and anxiety, lack treatment options [2, 3]. The main pathological hallmarks of ANDs are behavioral changes; excessive oxidative stress; progressive functional decline; impaired mitochondrial function; protein misfolding, aggregation, and accumulation; excitotoxicity; disturbed calcium (Ca^2+^) homeostasis; neuroinflammation; and neuronal cell death. Although these hallmarks are associated with neurodegeneration, they remain inconclusive [2, 4]. Moreover, recent evidence suggests that genetic mutation, environmental risk factors, and toxin exposure are important in chronic neurodegeneration pathogenesis [5, 6, 7]. According to the Global Burden of Disease Study, ANDs are a major burden on public health due to the increasing aged population, which is at high risk for several diseases [8, 9].

Only a few established drugs have been approved to treat ANDs, but they cannot prevent the disease from progressing [10]. The currently available AND drugs provide only symptomatic relief primarily by modulating neurotransmission [11]. Recently, molecular target-based therapies, such as direct receptor agonists and antagonists, neurotransmitter modulators, second messenger modulators, neurotrophic factors, stem cell-based therapies, hormone replacement therapy, and regulators of mRNA synthesis and translation into disease-causing mutant proteins, have been introduced and used [12]. Numerous traditional symptomatic therapies can lose their efficiency over time, produce disruptive symptoms of their own, and result in severe side effects [13]. Therefore, more effective, and safer therapeutic drugs are urgently needed to prevent or slow the progression of ANDs.

Natural products include various chemical compounds progressively selected for their ability to enhance an organism’s survival [14]. They have been extensively used in human health care as traditional medicines or dietary supplements for a millennium due to their various pharmacological activities [15]. Recently, natural products have emerged as promising AND drug candidates with multiple pharmacological activities on different validated targets. Natural products are likely to have a broader range of targets than chemical compounds, since they contain various compounds that differ in structure and biological activity [16, 17]. A systems-based approach showed that compounds derived from natural products are structurally more similar to human metabolites than conventional small-molecule drugs [18]. This systematic approach may provide evidence of the potential of natural products for multi-target activities. Therefore, natural products may be a promising therapeutic strategy for protecting and treating multifactorial diseases, such as ANDs [16, 17, 18].

## 2. Pathophysiological events in brain aging and the associated age-related neurological disorders

**Figure 1.**
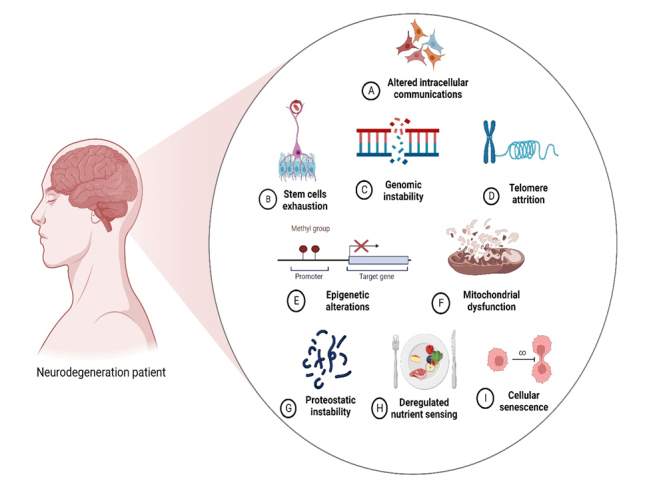
**Hallmarks of brain aging.** These include altered intercellular communication, stem-cell exhaustion, genomic instability, DNA damage, epigenetic changes, mitochondrial dysfunction, stress response, impaired protein quality control, deregulated nutrient sensing, and cellular senescence.

The brain biologically ages through cellular and molecular mechanisms. These aging-related dysfunctions are important risk factors for ANDs, including NDDs, and neuropsychological disorders, such as depression and anxiety [19]. However, an increasing number of studies suggest that oxidative stress, cell senescence, altered morphology, altered proteostasis, dysregulated neuronal Ca^2+^ homeostasis, hormones, altered gene expression, neurotransmitters, genetics, compromised DNA repair, energy metabolism dysfunction, mitochondrial dys-function, stem-cell exhaustion, autophagy impairment, and neuroinflammation may play a crucial role in brain aging [3, 20, 21]. When the body acts against harmful stimuli, such as pathogens, toxic molecules, damaged cells, and foreign bodies resulting from acute cellular stress or aging, it experiences low-level chronic inflammation. During chronic inflammation, inflammatory responses cause oxidative damage, resulting in decreased cortical volume and the progressive loss of the neuronal population, presynaptic markers, and dendritic spines, leading to cognitive dysfunction and memory loss [22]. However, biological aging might not progress as fast as chronological aging. Moreover, it may delay or prevent biological aging and even reduce the risk of age-related diseases such as dementia [23]. In particular, previous studies have reported that chemical exposure and environmental pollutants in the body can also cause cognitive impairment and memory decline [24], associated with symptoms such as depression, anxiety, hostility, and tension [24]. Hallmarks of brain aging, neurodegeneration, and ANDs are illustrated in Figure 1.

The brain is highly vulnerable to oxidative damage due to its relative insufficiency of antioxidant enzymes, such as glutathione peroxidase and catalase, and its abundance of oxidizable substrates, such as catecholamines and polyunsaturated fatty acids [25]. Much evidence suggests that the accumulation of different DNA (e.g., 8-hydroxy-deoxyguanosine [8-OH-dG]), protein (e.g., protein 3-nitrotyrosine and protein carbonyls), and lipid (e.g., lipid peroxidation products 4-hydroxynonenal and malondialdehyde [MDA]) oxidative damage markers lead to brain aging [26, 27]. In the context of lipid oxidation products and mitochondrial proteins, increased levels of 3-nitrotyrosine and carbonyls in proteins, fluorescent lipid peroxidation products, and thiobarbituric acid reactive substances and decreased protein thiol and cardiolipin content have been observed in aged rat brains [28, 29, 30]. Progressively increasing levels of 8-OH-dG have been observed in aged human brain tissue, which are 10-fold higher in mitochondrial DNA (mtDNA) than in nuclear DNA [31]. Similarly, increased 8-OH-dG levels in mtDNA than in nuclear DNA have also been observed in aged brains of six mammalian species [32]. Studies have shown that oxidative damage-induced mutations in mtDNA accumulate with age in postmitotic tissues such as the brain [33, 34, 35]. In addition, previous studies have shown that high mtDNA mutation levels are associated with ANDs [33, 35], with increased cortical mtDNA deletions found in AD patients [36]. Ikebe et al. found a 17-fold increase in mtDNA deletions in the striatum of PD patients compared with control subjects [37]. These findings indicate that mitochondrial bioenergetic function impairment in the aged brain is due to oxidative damage to mitochondrial lipid and protein components. However, mtDNA and nuclear DNA alterations due to oxidative damage can also contribute to the latter phenomenon by downregulating the levels of proteins involved in oxidative phosphorylation and mitochondrial transport systems.

The most important mitochondrial functional deficits in aged rodent, non-human primate, and human brains include a loss of mitochondrial membrane potential (MMP) and phosphorylation capacity and decreased adenosine triphosphate (ATP) synthesis, respiration, and mitochondrial permeability transition pore activation [28, 29, 38, 39]. Although several studies have found that respiratory complex activities are moderately decreased in the mitochondria of aged brains, the pattern and the extent of such decreases are variable [38, 39, 40]. However, a recent study reported no significant changes in brain mitochondrial respiration, respiratory chain activities, and ATP synthesis in 344 aged Fischer rats [41]. In addition, numerous studies have indicated an increase in complex IV activity in aged rodent brains [42, 43]. Few studies have evaluated the protein and mRNA levels of different subunits of F_0_F_1_-ATP synthase, respiratory chain proteins, or other mitochondrial proteins in the aged brain. However, the published results are somewhat inconsistent. One study found reduced protein levels of several subunits of complex I, complex IV, F_0_F_1_-ATP synthase, and adenine nucleotide translocase isoform 1 in aged rat brains. However, another study found no significant changes in the protein levels of cytochrome oxidase subunits (subunit I is encoded in the mtDNA and subunit IV in the nuclear DNA) in aged rat cerebellar cortexes [44, 45].

Several *in vitro* studies have shown that mitochondrial oxidative stress exposure leads to impaired functions in aged brain mitochondria. Such studies have suggested that oxidative stress is primarily responsible for the age-related decline in mitochondrial functions [46, 47]. Recently, a proteomic approach showed high levels of oxidative-modified mitochondrial proteins in aged brains, especially in the cortex and hippocampus, involved in energy metabolism, such as ATP synthase, aldolase, pyruvate kinase, α-enolase, and creatine kinase [48]. Finally, age-related alterations to the supramolecular assembly of respiratory complexes have been reported in rat-brain cortexes [49]. Although mitochondrial biogenesis could participate in the overall health benefits of mitochondrial function during brain aging, few studies have examined the status of nuclear respiratory factor 1, nuclear respiratory factor 2 (NRF2), peroxisome proliferator-activated receptor gamma coactivator 1-alpha, or mitochondrial transcription factor A. Similarly, few studies have reported enhanced reactive oxygen species (ROS) production by aged brain mitochondria. Moreover, few systematic studies have evaluated how antioxidant response element-dependent genes are activated by increased mitochondrial ROS production in aged brains [28].

Increasing evidence suggests that aging and ANDs are associated with inflammation [50, 51]. Microglia are the primary immune cells in the central nervous system (CNS). They constitutively express surface receptors that trigger or increase the innate immune response, including chemokine receptors, cytokine receptors, complement receptors, complex II, and the major histocompatibility complex [52]. During the cellular damage process, they respond quickly by inducing an immune response that potentially protects against pathogenic conditions by upregulating inflammatory molecules and neurotrophic factors [53]. However, chronic inflammation is attributed to microglia cell activation and their release of proinflammatory cytokines, such as interleukin 1β (IL-1β), tumor necrosis factor α (TNF-α), and interleukin 6 (IL-6). Elevated IL-1β, TNF-α, and IL-6 protein levels have been observed in the brains of aged animals [54, 55]. However, increased IL-6 protein levels in the hippocampus and cerebral cortex are primarily due to microglia in aged mouse brains [56]. Researchers propose that the increased brain microglial activation may be an early event in oxidative damage. Activated microglia are a rich source of free radicals in the brain and are responsible for releasing radicals such as nitric oxide and superoxide [57]. Microglia-derived radicals and their reaction products, i.e., peroxynitrite and hydrogen peroxide, can harm cells. These products are involved in oxidative damage and neuronal cell death in ANDs [58]. Interestingly, microglial cells have effective antioxidative protective mechanisms and contain high levels of glutathione (GSH), GSH reductase, GSH peroxidase, antioxidative catalase enzymes such as superoxide dismutase (SOD), and nicotinamide adenine dinucleotide phosphate-regenerating enzymes [58]. The endogenous antioxidant reserves become exhausted with excessive ROS production, resulting in cell damage.

## 3. Pharmacological importance of black pepper and its bioactive compounds

Herbs and spices have been an essential part of human nutrition since the dawn of humankind. They have been utilized for millennia to increase food flavor, color, and aroma and are recognized for their preservative and medicinal properties [59]. Black pepper belongs to the family Piperaceae and is widely cultivated in India, Vietnam, Malaysia, Indonesia, Thailand, Singapore, Philippines, and China [60, 61]. Black pepper is one of the most used spices in the world. It has a strong aromatic smell, making it suitable as a flavoring and seasoning agent in food. In addition to its culinary uses, its medicinal properties are used in traditional Chinese medicine and the ancient Indian medical system of Ayurveda to treat pain, asthma, bleeding, chills, rheumatism, flu, and muscular pain [62]. It is a good stimulant and a carminative, which is useful for treating diarrhea, constipation, cholera, gonorrhea, chronic malaria, tongue paralysis, and viral hepatitis. It is also commonly used to treat spleen diseases, bronchitis, and respiratory infections [63]. The fruits of black pepper have various biological activities, including antioxidant, antidiabetic, anticancer, antibacterial, analgesic, anti-inflammatory, anti-convulsant, and neuroprotective effects [64, 65].

Neuroprotective compounds isolated from black pepper are chemically characterized as pungent alkaloids, amide alkaloids, and alkamides. The major pungent compounds in black pepper are the amide alkaloid piperine and the alkaloids piperanine, piperlonguminine, pipercallosine, pipernonatine, dehydropipernonaline, guineensine, retrofractamide B, chabamide, retro-fractamide A, isopiperolein B, 6,7 dehydro-brachyamide B, paprazine, cepharadione A, piperolactam D, sylvamide, chavicine, and 10-tricosanone. Alkamides in black pepper include pellitorine, piperettine, piperettyline, feruperine, and piperine analogs such as HJ105, HJ22, and 3B. Table 1 lists black-pepper compound chemical structures and neuroprotective activities.

Alkamides compounds, such as pellitorine, piperettine, piperettyline, and feruperine, play major roles in black pepper’s various pharmacological actions. Neuroprotective, anticholinesterase, and antioxidant effects have been reported in black pepper, indicating that it has dual inhibitory activity against acetylcholinesterase (AChE), butyrylcholinesterase (BChE), and 2,2-diphenyl-1-picrylhydrazyl (DPPH) free-radical scavenging activity. In addition, it is the most potent selective BChE inhibitor and shows inhibitory activity for amyloid beta (Aβ) self-induced aggregation as an important disease-specific AD protein [74]. Recent *in vitro* and *in vivo* studies have shown that pellitorine could cross the Caco-2 cell monolayer, rapidly penetrating the gut and blood-brain barrier (BBB) to reach the brain parenchyma, indicating a possible role in the treatment of CNS diseases [70].

**Table 1:** Black-pepper compound chemical structures and neuroprotective activities.

Compound name	Study model	Neuroprotective	Ability to cross BBB	Other activities	References
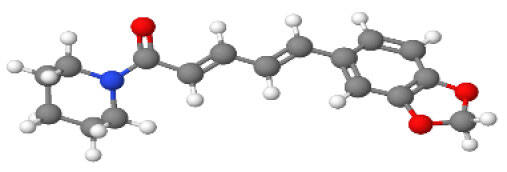 Piperine	*In vitro*/*in silico*	☑	☑	Can cross the BBB and has MAO-B inhibitory activity	[66, 67, 68]
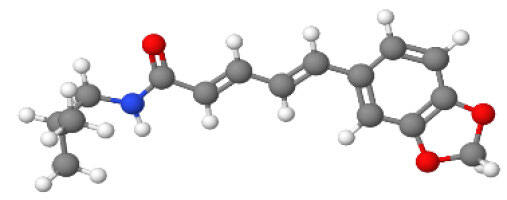 Piperlonguminine	*In vitro*/*in vivo*	☑	☑	Increased striatal dopamine levels and protective effects on mitochondrial complex I activity	[66, 69]
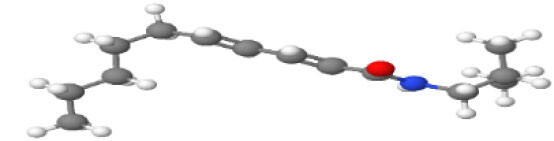 Pellitorine	*In vitro/in vivo*	☑	☑	Good gut permeation and can cross the BBB	[66, 70]
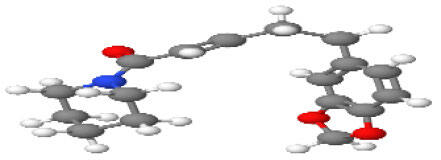 Piperanine	*In vitro*	☑	☑	Cytoprotective effects and low neurotoxicity in SH-SY5Y and SK-N-SH cells	[66]
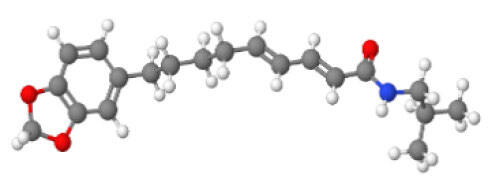 Pipercallosine	*In vitro*	☑	☑	Cytoprotective effects and low neurotoxicity in SH-SY5Y and SK-N-SH cells	[66]
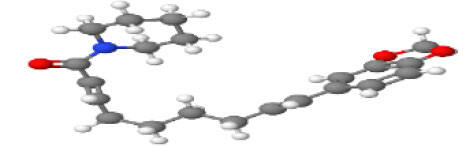 Pipernonatine	*In vitro*	☑	☑	Cytoprotective effects and low neurotoxicity in SH-SY5Y and SK-N-SH cells	[66]
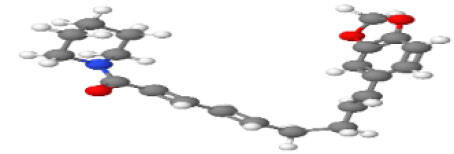 Dehydropipernonaline	*In vitro*	☑	☑	Cytoprotective effects and low neurotoxicity in SH-SY5Y and SK-N-SH cells	[66]
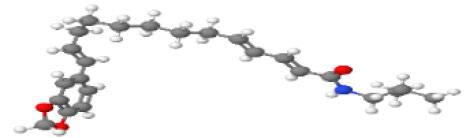 Guineensine	*In vitro*	☑	☑	Cytoprotective effects and low neurotoxicity in SH-SY5Y and SK-N-SH cells	[66]
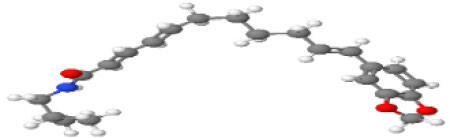 Retrofractamide B	*In vitro*	☑	☑	Cytoprotective effects and low neurotoxicity in SH-SY5Y and SK-N-SH cells	[66]
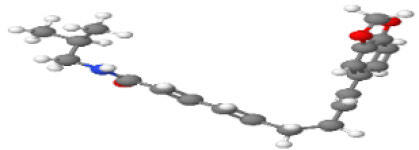 Retrofractamide A	*In vitro*	☑	☑	Has antioxidant and anti-inflammatory activities	[71]
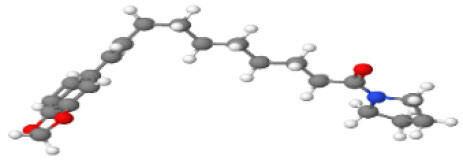 Isopiperolein B	*In vitro*	☑	☑	Has antioxidant and anti-inflammatory activities	[71]
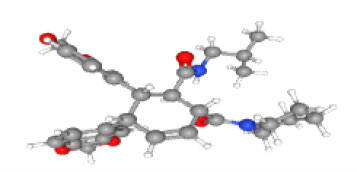 Chabamide	*In vitro*	☑	☑	Has antioxidant and anti-inflammatory activities	[71]
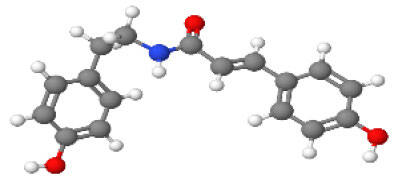 Paprazine	*In vitro/in vivo/in silico*	☑	☑	Has antidepressant, anxiolytic, antipyretic, and thrombolytic activities	[72]
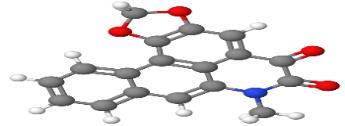 Cepharadione A	*In vitro/in vivo/in silico*	☑	☑	Has antidepressant, anxiolytic, antipyretic, and thrombolytic activities	[72]
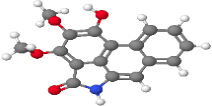 Piperolactam D	*In vitro/in vivo/in silico*	☑	☑	Has antidepressant, anxiolytic, antipyretic, and thrombolytic activities	[72]
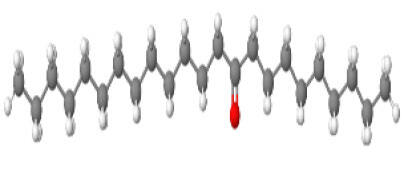 10-tricosanone	*In vitro/in vivo/in silico*	☑	☑	Has antidepressant, anxiolytic, antipyretic, and thrombolytic activities	[72]
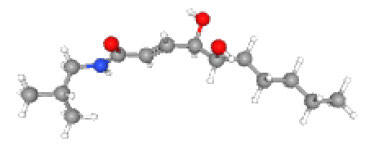 Sylvamide	*In vitro/in vivo/in silico*	☑	☑	Has antidepressant, anxiolytic, antipyretic, and thrombolytic activities	[72]
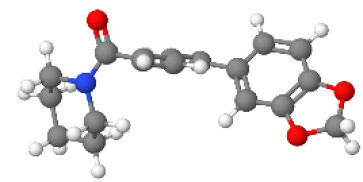 Chavicine	*In vivo*	☑	☑	Improved memory function and reduced oxidative damage	[73]
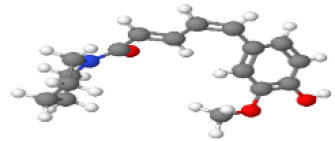 Feruperine	*In vitro/in silico*	☑	☑	Anti-AChE and BChE activities and potent DPPH free-radical scavenging activities	[74]
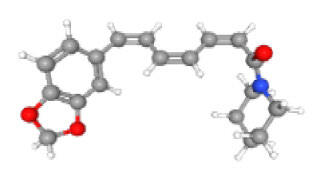 Piperettine	*In vitro/in silico*	☑	☑	Anti-AChE and BChE activities and potent DPPH free-radical scavenging activities	[74]
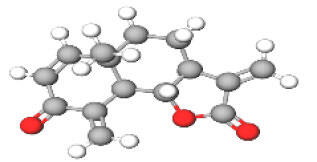 6,7 dehydrobrachyamide B	*In vitro/in silico*	☑	☑	Anti-AChE and BChE activities and potent DPPH free-radical scavenging activities	[74]
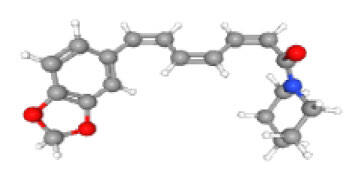 Piperettyline	*In vitro/in silico*	☑	☑	Anti-AChE and BChE activities and potent DPPH free-radical scavenging activities	[74]

In the ball and stick models, grey, red, white, and blue balls represent carbon, oxygen, hydrogen, and nitrogen, respectively. Key: ☑, has that ability:☑, does not have that ability; BBB, blood-brain barrier; MAO-B, monoamine oxidase-B; AChE, acetylcholinesterase; BChE, butyrylcholinesterase; DPPH, 2,2-diphenyl-1-picrylhydrazyl.

Piperine is the major pungent compound in black pepper and has various biological properties. It has been reported to ameliorate or prevent chronic diseases in human and animal models [75, 76]. Piperine is extensively used as a marker compound for the quality control of black-pepper extracts, raw materials, and commercial products [77, 78]. It also inhibits various metabolizing enzymes and increases the oral bioavailability of numerous drugs, nutrients, and vaccines by promoting their absorption through various mechanisms. Furthermore, it boosts cognitive actions and fertility. Piperine also modulates the membrane dynamics and increases the absorption-site permeability, crossing the BBB and showing monoamine oxidase B (MAO-B) inhibitory activity [67, 68, 79].

Piperlongumine is another major pungent ingredient in black pepper. Its pharmacological actions are varied, including antioxidant, anti-inflammatory, anticancer, neuroprotective, and anti-hyperlipidemic activities [80]. Piperlongumine can also cross the BBB, suppressing oxidative stress and age-related inflammation by inhibiting the mitogen-activated protein kinase (MAPK)/nuclear factor-κB (NF-κB) pathway [69, 81]. Amide alkaloids from black pepper, including dehydropipernonaline, guineensine, piperanine, pipernonatine, pipercallosine, and retrofractamide B, improve cell viability in the presence of neurotoxins, which suggests that black pepper’s amide alkaloids could increase neuroprotective effects, enhance bioavailability, and cross the BBB [66, 82, 83]. Four alkaloids (chabamide, retrofractamide A, isopiperolein B, and 6,7dehydrobrachyamide B) isolated from black-pepper fruits have shown antioxidative and anti-inflammatory activity [71]. Iqbal et al. observed that the alkaloid chavicine isolated from black pepper protected against aluminum chloride (AlCl_3_)-induced AD mice. Their results showed that chavicine treatment improved memory function and DPPH free-radical scavenging activity [73].

Another *in vitro* study showed the cognitive effects of HJ105, a piperine derivative, isolated from black pepper against Aβ_1-42_-induced SH-SY5Y cells. Interestingly, treatment with HJ105 (2, 10, or 50 μM) significantly improved cell viability by greatly suppressing Aβ_1-42_-induced apoptosis in SH-SY5Y cells. In addition, HJ105 treatment attenuated the increase in MDA and increased the activities of the antioxidant enzyme SOD, catalase, and plasma GSH peroxidase, activating Nrf2 by inhibiting the Keap1-Nrf2 interaction and markedly downregulating NLR family pyrin domain-containing 3 (NLRP3), PYD and CARD domain-containing, caspase-1, and IL-1β in SH-SY5Y cells exposed to Aβ_1-42_ [84]. In addition, piperine and its analog HJ22, isolated from black-pepper, showed potent therapeutic effects against ibotenic acid-induced cognitive impairment by reducing oxidative stress, cholinergic damage, apoptosis, and neuroinflammation in AD rats [85]. Piperine analog 3B (12.5, 25, 50, and 100 μM), isolated from black-pepper fruits, showed cytoprotective effects by suppressing ROS accumulation and restoring MMP in PC12 cells. This activity might be related to Nrf2 activation and the expression of corresponding antioxidant proteins by promoting Nrf2’s nuclear entry, activating cellular oxidative stress and protecting PC12 cells. An *in vivo* study showed that oral administration (100 mg/kg/body weight [b.w.]) of 3B significantly improved motor behavior and rescued dopaminergic neuronal cell death in the 1-methyl-4-phenyl-1,2,3,6-tetrahydropyridine (MPTP)-induced PD mice model [86].

Interestingly, a computational study using a molecular docking system suggested that black-pepper compounds have a multi-target ligand-binding ability. For example, piperine contains C=O, two R-O-R, and C-OH groups in its benzodioxole subunits, enabling it to form more hydrophobic interactions with amino acids in the α-amylase and α-glucosidase binding sites, which thereby ensures greater enzyme inhibitory activity and antioxidant properties [87]. Manap et al. performed a molecular docking study using the AutoDock Vina software. They found that piperine formed five hydrophobic interactions and contained many hydrogen bond donor and acceptor groups at the meta or para position, giving it essential inhibitory activity against AChE and Aβ aggregation [88]. Emon et al. performed an antidepressant, anxiolytic, and thrombolytic docking analysis with two receptors, i.e., human serotonin transporter and a potassium channel. They found that eight bioactive compounds (cepharadione A, piperolactam D, diazepam, paprazine, piperine, 10-tricosanone, sylvamide, and pellitorine) isolated from the methanolic black-pepper extract were primarily responsible for its antidepressant, anxiolytic, and thrombolytic activity by interacting with said target proteins with docking scores of -3.08-7.14, -1.0-7.9, and -4.5-7.9 kcal/mol, respectively [72]. Therefore, structure-activity relationships show that black-pepper compounds have potential neuroprotective effects by targeting different ligand sites. Indeed, this review shows that the most studied neuroprotective bioactive compound, i.e., piperine, has pharmacological actions and mechanisms in various ANDs.

## 4. Neuroprotective effects of black pepper and piperine in age-related neurological disorders: Preclinical studies

**Figure 2.**
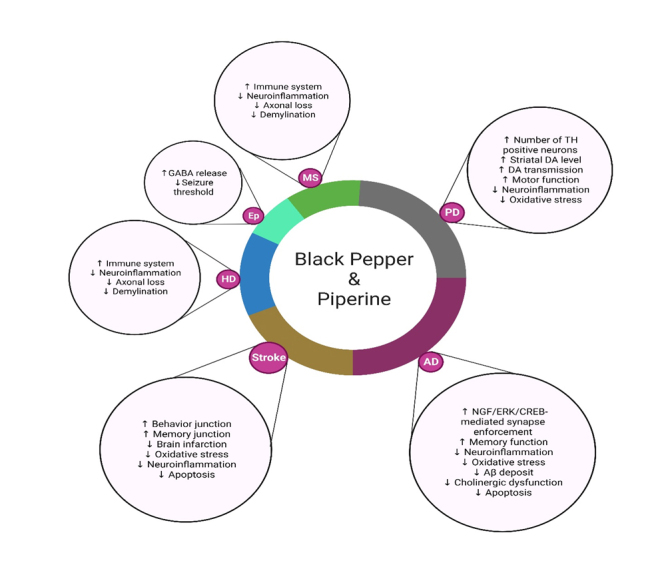
**Neuroprotective effects of black pepper and its constituent piperine in age-related neurological disorders.** Black pepper affects AD, PD, HD, MS, ischemic stroke, and epilepsy through each underlying mechanism. Down-arrows (↓) and up-arrows (↑) indicate inhibition and activation by black-pepper treatment, respectively. Key: Aβ, amyloid beta; DA, dopamine; SOD, superoxide dismutase; HO-1, heme oxygenase; Nrf2, nuclear factor erythroid 2-related factor 2; GABA, γ-aminobutyric acid; TH, tyrosine hydroxylase.

This section discusses the therapeutic potential of black pepper and its bioactive constituent, i.e., piperine, to provide neuroprotective effects in various neurological disorders, including AD, PD, MS, HD, epilepsy, ischemic stroke, depression, and anxiety, and to protect against neuroinflammation and neuronal cell death. The recent experimental findings of black pepper and piperine on ANDs are summarized in Figure 2 and Table 2.

### 4.1 Black-pepper activity in Alzheimer’s disease

AD is the most common neurodegenerative disorder in older adults and is currently a major public-health problem [89]. A neuropathological hallmark exhibited in AD brains is the excessive accumulation of neurotoxic Aβ peptides and abnormally hyper-phosphorylated microtubule-associated protein Tau [90]. This process ultimately causes synaptic dysfunction, subsequent neuronal loss, and atrophy of the cortex and hippocampus [91]. Current clinical AD treatments mainly target symptoms, and disease-modifying pharmacologic treatments are very limited.

The neuroprotective role of dietary supplements has been extensively demonstrated via beneficial effects on cognition and AD neuropathology, and thus they potentially represent a safe and natural AD treatment. In this regard, the antioxidant potential of black pepper and its bioactive molecules is of considerable importance for improving brain function and preserving cognition in AD. However, black-pepper fruit-extract treatment (35 μg/mL) has been shown to downregulate the pro-apoptotic phosphorylated forms of proteins glycogen synthase kinase 3 beta (p-GSK3β), RELA proto-oncogene NF-κB subunit (p-RELA/p-P65), and MAPK 8 (p-MAPK8/p-JNK) while upregulating MCL1 apoptosis regulator Bcl2 family member (MCL1), Bcl2-like 1 (Bcl2L1/Bcl-xl), and survivin; however, it does not appear to affect caspase-3 and -9 activation. In addition, black-pepper fruit extract has been shown to inhibit AChE function and Aβ aggregation in hydrogen peroxide (H_2_O_2_)-induced SH-SY5Y cells [92]. Manap et al. observed that combining piperine and curcumin preserved cell viability by up to 85%, indicating greater AChE inhibition in Aβ-induced SH-SY5Y cells. Moreover, combining piperine and curcumin at 35 µM significantly reduced MDA levels and significantly elevated GSH levels and catalase activity, inhibiting Aβ_42_ fibrillation and disaggregating the fibril formed in Aβ-induced SH-SY5Y cell [88].

**Table 2:** Detailed experimental studies of black pepper and its bioactive compound piperine on age-related neurological disorders.

Cell line/animal	Neurotoxin	Drug concentration	Mechanisms of action of black pepper and piperine	Reference
**Alzheimer’s disease**				
SH-SY5Y cells	H_2_O_2_ (250 µM for 24 h)	Black-pepper fruits (35 µg/mL)	Black pepper increased cell viability. It had antiapoptotic effects, increasing MCL1 and BCL-xl expression and inhibiting p-GSK3β, p-P65, and p-JNK, which ultimately inhibited AChE function and Aβ aggregation	[92]
Male albino rats	AlCl_3_ (17 mg/kg b.w. for two months)	Black pepper (20 and 200 mg/kg b.w. for two months)	Black pepper decreased AChE levels, inhibited amyloid plaque formation, and increased memory function and movement behavior	[93]
Male C57BL/6 mice	Immobilized stress (for two weeks)	Black-pepper fruits (100 and 200 mg/kg b.w. for two weeks)	Black pepper significantly inhibited MDA levels and AChE activity; increased catalase, SOD, and GSH activities; and improved motor function and learning ability	[94]
Sprague-Dawley rats	AlCl_3_ (17 mg/kg b.w. for one month)	Black pepper (93.75 and 187.5 mg/kg b.w. for three months)	Black pepper downregulated AChE activity, C-reactive protein, and monocyte chemoattractant protein-1; decreased NF-κB expression; and significantly increased ACh levels	[95]
Male Wistar rats	Aβ_1-42_ (4 µL)	Black pepper (50 and 100 mg/kg b.w. for 21 days)	Black pepper significantly inhibited MDA levels and AChE activity; increased catalase, SOD, and GSH activities; and improved motor function and memory performance	[96]
Male C57BL/6 mice	AlCl_3_ (250 mg/kg b.w. for 30 days)	Black pepper (12.5 mg/kg/b.w. for 30 days)	Black pepper decreased amyloidogenic APP_770_ levels, improved non-amyloidogenic APP_695_ levels, and enhanced memory function	[73]
Male Swiss albino mice	Scopolamine (0.75 mg/kg b.w. for 14 days)	Black pepper ([Viphyllin] 50 and 100 mg/kg/b.w. for 14 days)	Black pepper dose-dependently improved spatial memory and cholinergic function; inhibited oxidative damage and histopathological changes; attenuated the upregulation of MAPK/p-p38, MAPK/p-JNK, Bax/Bcl-2 ratio, and caspases activation; downregulated COX-2, TNF-α, and NOS-2; markedly reduced the proBDNF/mBDNF ratio; and upregulated TrkB expression	[97]
Male Wistar rats	Ethylcholine aziridinium ion (4 µL for one week)	Piperine (5, 10, and 20 mg/kg b.w. for two weeks)	Piperine improved memory function and increased hippocampal neuron density, decreased cognitive deficits, and inhibited AChE and MDA activity	[98]
Male C57BL/6 mice	Streptozotocin (1.5 mg/kg b.w. for one, three, and eight days)	Piperine (2.5, 5, and 10 mg/kg b.w. for 15 days)	Piperine ameliorated cognitive impairments and enhanced memory function and movement performance; restored hippocampal neurotransmission (norepinephrine, DA, serotonin, GABA, and glutamate); attenuated increased levels of MDA activity, NO, IL-1β, IL-6, and TNF-α; and upregulated anti-inflammatory cytokines IL-14 and IL-10	[99]
**Parkinson’s disease**				
SK-N-SH and SH-SY5Y	6-OHDA (100 μM for SK-N-SH cells and 500 μM for SH-SY5Y cells for 24 h)	Black-pepper fruits (5, 10, 20, and 40 μg/mL for 24 h)	Black pepper showed potential neuroprotective effects, improved cell viability, and suppressed 6-OHDA-induced cell injuries	[66]
SK-N-SH	SNCA (transfection for 24 h)	Piperine (0.2, 1, 5, 25, and 125 μM for 24 h)	Piperine treatment improved cell viability, reduced cytotoxicity by promoting SNCA degradation, blocked autophagy flux, and inhibited SNCA overexpression via P2RX4 activation	[100]
SK-N-SH	Rotenone (100 nM for 24 h)	Piperine (1, 5, and 25 μM for 24 h)	Piperine treatment enhanced cell viability, reduced rotenone-induced cytotoxicity, maintained mitochondrial function, enhanced mitochondrial complex I activity, downregulated p-S6K levels by suppressing mTORC1, and induced autophagy activation	[101]
Male Wistar rats	6-OHDA (10 µg/kg b.w. for one day)	Piperine (10 mg/kg b.w. for 15 days)	Piperine improved motor coordination and balance behavior, suppressed oxidative stress, provided antiapoptotic effects, and attenuated inflammation by downregulating IL-1β and TNF-α	[102]
Male C57BL/6 mice	MPTP (30 mg/kg b.w. for seven days)	Piperine (10 mg/kg b.w. for 15 days)	Piperine attenuated MPTP-induced impairments in motor coordination and cognitive function, prevented the decrease in the TH-positive neurons in the substantia-nigra region, suppressed oxidative damage, reduced the number of activated microglia, reduced IL-1β and Iba-1 expression, and had antiapoptotic effects by maintaining the Bax/Bcl-2 balance	[103]
Thy 1-*Snca* TG and CAG-RFP-EGFP-LC3 mice	-	Piperine (25, 50, and 100 mg/kg b.w. for six weeks)	Piperine alleviated olfactory deficits and improved motor function in *Snca* transgenic mice by increasing striatal DA levels and promoting SCNA degradation and aggregation	[100]
Male C57BL mice/SK-N-SH cells	Rotenone (30 mg/kg b.w. for six weeks)	Piperine (25 mg/kg b.w. for four weeks)	Piperine attenuated motor deficits by inducing striatal DA levels and increasing TH-positive neurons	[101]
**Huntington’s disease**				
Male Wistar rats	3-NP (20 mg/kg b.w. for four days)	Piperine (10 mg/kg b.w. twice daily for four days)	Piperine improved neurobehavioral function, restored 5-HT levels, increased MAO activity, reduced neuron degeneration in striatal tissue, significantly reduced GFAP immunoreactivity, and prevented neuronal dysfunction and death	[104]
Male Wistar rats	Quinolinic acid (200 nmol/2 μL for seven days)	Piperine (2.5 mg/kg b.w.) in combination with curcumin (25 and 50 mg/kg b.w. daily for 21 days)	Piperine combined with curcumin restored body weight and attenuated behavioral performance induced by quinolinic acid. It also suppressed oxidative damage; inhibited NO levels; decreased inflammatory cytokines IL-1β, IL-6, and TNF-α; and increased neurochemical striatal levels	[105]
Male Wistar rats	3-NP (10 mg/kg b.w. for 21 days)	Piperine (2.5 mg/kg b.w) in combination with curcumin (25 and 50 mg/kg b.w. daily for 21 days)	Piperine combined with curcumin positively modulated behavioral performance; suppressed oxidative damage; significantly decreased IL-1β and TNF-α levels; and altered neurochemical levels	[106]
**Multiple sclerosis**				
Lewis rats	EAE animal model	Piperine (5 mg/kg b.w. for four weeks)	Piperine had neuroprotective and antiapoptotic effects in EAE by reducing caspase-3 and enhancing BDNF- and NeuN-expressing cells; it decreased levels of proinflammatory cytokines TNF-α, IL-1β, and iNOS; enhanced IL-10, Nrf2, HO-1, and MBP protein levels; enhanced total antioxidant capacity; and reduced oxidative damage in the CNS	[107]
Lewis rats	Bilateral injection of lysolecithin (2 μL)	Piperine (5, 10, and 20 mg/kg b.w. for 13 days)	Piperine improved memory performance and reduced myelin repair in the hippocampus; inhibited iNOS expression by upregulating the expression of TNF-α, IL1-β, and NF-κB; increased glial activation in the injured area; upregulated Nrf2 and HO-1 expression; enhanced total antioxidant capacity; and upregulated IL-10, Foxp3, BDNF, and MBP expression in hippocampal tissue	[108]
**Epilepsy**				
Male Sprague-Dawley rats	Pilocarpine hydrochloride (340 mg/kg) with methyl scopolamine (1 mg/kg b.w. for 45 days)	Piperine (40 mg/kg b.w. for 45 days)	Piperine reduced status epilepticus; prevented memory impairment; reduced oxidative damage; showed antiapoptotic effects by downregulating caspase-3 activity and increasing Bcl-2 protein levels; and decreased the expression of proinflammatory cytokines TNF-α and IL1-β	[109]
Male Swiss albino mice	Maximal electroshock	Piperine (5, 10, and 20 mg/kg b.w. for 30 mins)	Piperine increased GABA and serotonin levels in the hippocampus and cortex	[110]
Male Swiss mice	Pilocarpine (350 mg/kg b.w. for 30 mins)	Piperine (2.5, 5, 10, and 25 mg/kg b.w. for 30 mins)	Piperine increased striatal levels of GABA, glycine, and taurine; attenuated pilocarpine-induced increases in nitrite contents in the sera and brain; and reduced immunostainings for TNF-α in all regions (CA1, CA3, and DG) of the hippocampus and cortex	[111]
Albino rats	Pilocarpine (350 mg/kg b.w. for 30 mins)	Piperine (25 mg/kg b.w. for 10 days)	Piperine treatment significantly increased serotonin levels in the brain due to its MAO inhibitory activity	[112]
**Ischemic stroke**				
Male Sprague-Dawley rats	pMCAO	Black-pepper fruits (100 and 200 mg/kg b.w. for 14 days)	Black pepper improved behavioral performance; reduced cellular damage by decreasing the number of shrunken neuronal cells; increased neuronal density; increased PSD-95 and SYN-I protein levels; decreased α-syn immunoreactivity in the brain; and increased PSD-95, p-CaMK II, CaM, and NR2B levels	[113]
Male Sprague-Dawley rats	pMCAO	Black-pepper fruits (50, 100, and 150 mg/kg b. w. for 14 days)	Black pepper reduced infarct volume, neurological score, and brain damage; downregulated ATG7 and p-AKT; and upregulated p-mTOR	[114]
Male Sprague-Dawley rats	pMCAO	Piperine (10 and 20 mg/kg b.w. for 14 days)	Piperine improved behavioral performance; reduced cellular damage, areas of cerebral infarction, and major macro- and micro-cellular cerebral structural changes; inhibited BAX; decreased caspase-3, caspase-9, and cytochrome-c expression; and increased Bcl-2 expression	[115]
Male Sprague-Dawley rats	Ischemia/reperfusion injury	Piperine (20 mg/kg b.w. for 15 days)	Piperine treatment reduced infarct volume and cortical neuronal loss and significantly decreased complement component 3, fibrinogen gamma chain, alpha-2-macroglobulin, and transferrin protein levels	[116]
Male Sprague-Dawley rats	MCAO	Piperine (10 mg/kg b.w. for 15 days)	Piperine treatment reduced infarct volume and motor dysfunction; suppressed oxidative damage by reducing LPO and increasing GSH levels; preserved mitochondrial function; enhanced mitochondrial complex I activity; decreased expression of Bax, caspase-3, caspase-9, and cytochrome-c; increased Bcl-2 expression; increased BDNF and CREB expression; and decreased IL1-β and GFAP expression	[117]
Male Wistar rats	MCAO	Piperine (10 mg/kg b.w. for 15 days)	Piperine treatment significantly improved behavior; reduced levels of proinflammatory cytokines IL1-β, IL-6, and TNF-α; and reduced COX-2, NOS-2, and NF-κB expression	[118]
Male Sprague-Dawley rats	pMCAO	Piperine (10, 20, and 30 mg/kg b.w. for 14 days)	Piperine treatment reduced infarct volume; improved behavior function; increased neural survival; reduced cerebral cortex and striatum damage; increased PI3K expression; and significantly decreased p-PI3K, p-AKT, and p-mTOR levels	[119]
HT22 cells	Oxygen-glucose deprivation	Piperine (20, 30, and 40 μg/mL)	Piperine remarkably increased cell viability, increased PI3K expression, and significantly decreased p-PI3K, p-AKT, and p-mTOR levels	
**Depression and anxiety**				
Male ICR mice	UCMS (for six weeks)	Black-pepper fruits combined with Kleeb Bua Daeng (100 and 500 mg/kg b.w. for three weeks)	Black pepper with Kleeb Bua Daeng treatment reduced motor deficits; promoted neurogenesis by upregulating BDNF and CREB expression; and suppressed TNF-α, IL-1β, and IL-6 expression in the frontal cortex and hippocampus	[120]
Male ICR mice	UCMS (for six weeks)	Black-pepper fruits combined with Kleeb Bua Daeng (100 and 500 mg/kg b.w. for three weeks)	Black pepper with Kleeb Bua Daeng treatment improved memory function, decreased serum corticosterone and MDA levels, and increased catalase and SOD activities in the frontal cortex and hippocampus	[121]
Male ICR mice	Corticosterone (5 mL/kg b.w. for three weeks)	Piperine (10 mL/kg b.w. for three weeks)	Piperine increased sucrose intake, significantly attenuated the increase in FST test immobility time, and increased BDNF expression	[122]
Sprague-Dawley rats	UCMS (for five weeks)	Piperine (10 mg/kg b.w. for five weeks)	Piperine increased sucrose intake, significantly attenuated the increase in FST test immobility time, and increased BDNF expression in the hippocampus and frontal cortex	[123]

Key: Aβ, amyloid beta; ACh, acetylcholine; APP, Aβ precursor protein; b.w., body weight; DA, dopamine; SOD, superoxide dismutase; AChE, acetylcholinesterase; HO-1, heme oxygenase; Nrf2, nuclear factor erythroid 2-related factor 2; GABA, γ-aminobutyric acid; TH, tyrosine hydroxylase; NF-κB, nuclear factor kappa B; ROS, reactive oxygen species; iNOS, inducible nitric oxide synthase; COX-2, cyclooxygenase-2; Bcl2, B-cell lymphoma 2; BDNF, brain-derived neurotrophic factor; PSD-95, postsynaptic density protein 95; MDA, malondialdehyde; GSH, glutathione; GPx, glutathione peroxidase; FST, forced swim test; TST, tail suspension test; IL-1β, interleukin-1β; IL-6, interleukin-6; TNF-α, tumor necrosis factor-α; GFAP, glial fibrillary acidic protein; IBA-1, ionized Ca^2+^-binding adapter molecule 1; H_2_O_2_, hydrogen peroxide; AlCl_3_, aluminum chloride; 6-OHDA, 6-hydroxydopamine; MPTP, 1-methyl-4-phenyl-1,2,3,6-tetrahydropyridine; 3-NP, 3-nitropropionic acid; EAE, experimental autoimmune encephalomyelitis; MCAO, middle cerebral artery occlusion; UCMS, unpredictable chronic mild stress; NO, nitric oxide; 5-HT, 5-hydroxytryptamine.

The oral black-pepper supplementation (20 or 200 mg/kg/b.w.) provided neuroprotection, including remarkable decreases in cholinesterase levels and amyloidal plaque formation, and increased memory function in AlCl_3_-induced AD rats [93]. Rashedinia et al. previously investigated the anti-AD effect of aqueous black-pepper fruit extracts (100 and 200 mg/kg/b.w.). Their results showed that aqueous black-pepper fruit extracts significantly reduced MDA levels and AChE activity; increased catalase, SOD, and GSH activities; and improved motor function and learning ability in the immobility stress-induced AD mouse model [94]. Ahmed et al. subsequently reported that black pepper (93.75 or 187.5 mg/kg/b.w.) treatment significantly reduced serum and brain AChE activity, C-reactive protein levels, and monocyte chemoattractant protein-1 levels but significantly increased acetylcholine (ACh) levels in AlCl_3_-induced AD rats [95]. Additionally, Hritcu et al. reported that methanolic black-pepper fruit extract (50 and 100 mg/kg/b.w.) significantly improved memory performance and ameliorated Aβ_1-42_-induced spatial-memory impairment by enhancing antioxidant enzyme levels and attenuating oxidative stress in the hippocampus of a rat AD model [96]. Similarly, oral administration of 12.5 mg/kg/b.w. of black pepper to AlCl_3_-induced mice significantly improved memory function and decreased the Aβ precursor protein (APP) APP_770_ (amyloidogenic) isoform but increased the APP_695_ (non-amyloidogenic) isoform in the amygdala, cortex, and hippocampus. In addition, chavicine (a black-pepper derivative) significantly improved contextual memory function, showing a prominent effect on the hippocampus and amygdala and rescuing the APP ratios [73]. Sudeep et al. tested supercritical fluid extraction of black-pepper seeds, standardized to contain 30% β-caryophyllene (Viphyllin), in the scopolamine-induced dementia mouse model. They found that Viphyllin administration (50 or 100 mg/kg/b.w.) had neuroprotective effects by dose-dependently protecting against oxidative damage and histopathological changes while improving recognition, spatial memory, and cholinergic functions. In addition, Viphyllin (100 mg/kg/b.w.) significantly attenuated scopolamine-induced upregulation of p-JNK and p-p38 MAPK proteins, BAX/BCL-2 ratio, and caspase activation in the brain. Moreover, Viphyllin exerted anti-inflammatory effects by downregulating COX-2, TNF-α, and nitric oxide synthase 2 (NOS-2); markedly reducing the proBDNF/mBDNF ratio; and upregulating TrkB expression [97].

Chonpathompikunlert et al. investigated the effect of piperine, the main bioactive molecule in black-pepper fruits, on memory performance and neuronal apoptosis in an animal AD model. They found that piperine treatment (5, 10, or 20 mg/kg/b.w.) improved memory function and decreased cognitive deficiency. The underlying mechanism was partially associated with increased neuron density and inhibition of AChE and MDA activity in the hippocampus of ethylcholine-aziridinium-ion-induced AD rats [98]. More recently, piperine treatment (2.5, 5, or 10 mg/kg/b.w.) ameliorated streptozotocin-induced cognitive impairment and restored hippocampal neurotransmission (e.g., norepinephrine, dopamine [DA], serotonin, γ-aminobutyric acid [GABA], and glutamate levels). In addition, piperine attenuated the increase in MDA activity and nitric oxide (NO), IL-1β, IL-6, and TNF-α levels and significantly upregulated anti-inflammatory cytokines IL-4 and IL-10 in the hippocampus of streptozotocin-infused AD mice [99].

### 4.2 Black-pepper activity in Parkinson’s disease

PD is the most common movement disorder, mainly induced by progressive damage to dopaminergic neurons in the substantia-nigra pars compacta and DA depletion in the striatum, which is associated with motor impairments and poor cognitive performance in PD [124]. Interestingly, α-synuclein accumulation has been broadly associated with several neurotoxin pathways, including phospholipids, posttranslational modifications, mitochondrial dysfunction, endoplasmic reticulum stress, oxidative stress, synaptic dysfunction, altered mitochondrial morphology, neuroinflammation, and metal ions [4, 125]. Recent studies have suggested a proportional relationship between consuming a diet rich in natural products and improved health outcomes, including reduced PD risk.

Recent pharmacological animal and *in vitro* studies have shown that black pepper and its active ingredient, i.e., piperine, might be useful in treating PD. Yu et al. recently reported that black-pepper extracts, including fruits, pericarp, and leaves, improve cell viability and have potential neuroprotective activity in 6-hydroxydopamine (6-OHDA)-induced SK-N-SH and SH-SY5Y cells [66]. Recent *in vitro* results showed that piperine treatment improves cell viability and reduces cytotoxicity by promoting synuclein alpha (SNCA) degradation in SK-N-SH cells. In addition, the treatment protected SK-N-SH cells against blocked autophagy flux due to SNCA overexpression by activating purinergic receptor P2X 4 (P2RX4). These protective effects were exerted via autophagy flux promotion by enhancing autophagosome-lysosome membrane fusion [100]. Liu et al. observed that piperine treatment increased cell viability, restored mitochondrial function, and enhanced mitochondrial complex I activity. In addition, piperine induced autophagy by inhibiting mammalian target of rapamycin complex 1 (mTOR) via activation of protein phosphatase 2A in SK-N-SH cells and primary neurons [101].

Saeri et al. reported that the black-pepper neuroprotective effect combined with *Cyperus rotundus*, *Crocus sativus*, and *Boswellia serrata* effectively improves cognitive impairment induced by thyroid hormone deficiency in rats, probably by protecting brain tissue against oxidative damage [126]. Shrivastava et al. subsequently strengthened piperine’s (10 mg/kg/b.w.) neuroprotective and anti-inflammatory effects through *in vivo* trials. Their results showed that piperine improves motor function and balance behavior and suppresses oxidative stress in 6-OHDA-induced PD rats. In addition, piperine effectively inhibited apoptosis by blocking cytochrome-c, caspase-3, and caspase-9 release; maintaining the Bcl-2/Bax ratio; preventing poly(ADP-ribose) polymerase overactivation; and inhibiting inflammation by abrogating cytokine, TNF-α, and IL-1β levels in 6-OHDA-induced PD rats [102]. Similarly, Sharma et al. reported that piperine was a potential natural source of antioxidants. Therefore, the presence of these functional ingredients in piperine (2.5 mg/kg/b.w.) combined with quercetin (25 or 50 mg/kg/b.w.) makes it a strong candidate for ameliorating oxidative stress and improving behavioral function (e.g., open field, rotarod, narrow-beam walk, and grip strength tests). In addition, piperine with quercetin significantly enhanced neurotransmitter levels (e.g., DA, norepinephrine, serotonin, GABA, and glutamate), improved mitochondrial complex I and IV activities, and attenuated levels of proinflammatory cytokines TNF-α, IL-6, and IL-1β in rotenone-induced PD rats [127].

Yang et al. found that piperine treatment (10 mg/kg/b.w.) attenuated MPTP-induced deficits in motor coordination and cognitive function. Their results showed that piperine treatment augmented MPTP-induced decreases in tyrosine hydroxylase (TH)-positive cells in the substantia nigra, decreased oxidative stress and IL-1β expression, and reduced the number of activated microglia following MPTP treatment. Piperine’s antiapoptotic activity was shown by maintaining the balance of Bcl-2/Bax in MPTP-injected PD mice [103]. A recent animal study showed that piperine treatment (25, 50, or 100 mg/kg/b.w.) attenuated olfactory deficits and delayed motor deficits in Thy 1-*SNCA* transgenic mice. These protective effects were exerted by promoting autophagy flux via enhanced autophagosome-lysosome membrane fusion [100]. Similarly, piperine treatment (25 mg/kg/b.w.) attenuated rotenone-induced motor deficits, increased striatal DA levels, and rescued dopaminergic neuron loss in the substantia-nigra region [101].

### 4.3 Black-pepper activity in Huntington’s disease

HD is a complex neurodegenerative disorder characterized by motor-function disability and mental irregularity due to progressive degeneration of striatal neurons [128]. Oxidative stress and mitochondrial dysfunction may also provoke HD due to ROS production, which can lead to protein misfolding, which, in turn, causes inclusion bodies to form that clump together and stop neurotransmission [129]. Current therapeutic approaches suppress symptom severity and do not cure HD completely [130].

Salman et al. found that piperine (10 mg/kg/b.w.) protected 3-nitropropionic acid (3-NP)-treated rats by restoring 5-hydroxytryptamine (5-HT) levels, improving their neurobehavioral performance (rotarod, narrow-beam walk, gait analysis, grip strength, and elevated plus maze test), enhancing their monoamine oxidase (MAO) activity, and reducing neuron degeneration in striatal tissue. It has also been reported that piperine treatment significantly reduces glial fibrillary acidic protein (GFAP) immunoreactivity and prevents neuronal dysfunction and death in toxin-insulted animals [104]. Two studies reported that curcumin (25 or 50 mg/kg/b.w.) combined with piperine (2.5 mg/kg/b.w.) had beneficial effects on motor deficits and biochemical (LPO, nitrite, and GSH) and neurochemical (DA, norepinephrine, GABA, glutamate, 5-HT, 3,4-dihydroxyphenylacetic acid [DOPAC], and homovanillic acid [HVA]) abnormalities in 3-NP- and quinolinic acid-induced HD rats. Furthermore, combining piperine with curcumin significantly reduced the expression of proinflammatory cytokines such as IL-1β, IL-6, and TNF-α in quinolinic acid-induced [105] and IL-1β and TNF-α in 3-NP-induced [106] HD rats.

### 4.4 Black-pepper activity in multiple sclerosis

MS is the most common chronic immune-mediated NDD of the CNS. Axonal loss, inflammation, and demyelination accompany the early MS phase, which is affected by oxidative stress and metabolic, genetic, and environmental factors [131]. Rats were immunized with a suspension comprising guinea-pig spinal-cord homogenized in complete Freund’s adjuvant and toxin to induce experimental autoimmune encephalomyelitis (EAE). Lumbar spinal-cord cross sections showed that piperine (5 mg/kg/b.w.) significantly reduced inflammation, immune cell infiltration, demyelination, microglia, and astrocyte activation. Gene expression analysis in the lumbar spinal cord showed that piperine treatment enhanced IL-10, Nrf2, HO-1, and MBP expression but decreased levels of proinflammatory cytokines TNF-α, IL-1β, and inducible nitric oxide synthase (iNOS). Piperine treatment also enhanced the total antioxidant capacity and BDNF- and NeuN-expressing cells but reduced MDA levels and caspase-3 activity [107]. Similarly, Roshanbakhsh et al. found that piperine treatment (5, 10, or 20 mg/kg/b.w.) improved memory performance and myelin repair in the hippocampal demyelination model. Piperine also enhanced total antioxidant capacity and Nrf2 and HO-1 expression in hippocampal tissue. However, FOXP3, BDNF, and MBP expression were significantly increased [108]. Another study reported that piperine treatment (10 or 30 mg/kg/b.w.) had strong preventive and therapeutic effects on myelin oligodendrocyte glycoprotein-induced allergic encephalomyelitis by inhibiting inflammatory-cell infiltration into the CNS and preventing myelin degradation and BBB disruption in the EAE mouse model. In addition, piperine directly interacted with dihydroorotate dehydrogenase, inhibiting its enzymatic activity, greatly impairing concanavalin A-induced T lymphocyte proliferation, and suppressing mixed lymphocyte activities in a dose-dependent manner [132].

### 4.5 Black-pepper activity in epilepsy

Epilepsy is an incurable neurological disorder characterized by chronic and persistent neuronal activity due to a reduced seizure threshold in the CNS, affecting ~50 million people worldwide. Among the suggested underlying mechanisms, the imbalance between inhibitory GABA-mediated and excitatory glutamate-mediated neurotransmission is associated with behavioral abnormalities and brain dysfunction [133].

Several studies have reported the therapeutic effectiveness of essential oils as an alternative to the currently available drugs for managing epilepsy [134]. The anticonvulsant activities of piperine, a bioactive constituent of black pepper, on memory performance, oxidative stress, and inflammation have been investigated in a pilocarpine-induced rat epilepsy model. These results showed that piperine supplementation (40 mg/kg/b.w.) reduced status epilepticus and memory impairment by enhancing catalase and SOD activities and GSH content but reducing MDA levels. Piperine also decreased the levels of proinflammatory cytokines TNF-α and IL-1β, caspase-3 activity, and Bax and Bcl-2 expression in the pilocarpine-induced rat epilepsy model [109]. Additionally, piperine’s anticonvulsant effect has also been studied. Mishra et al. observed that piperine treatment (5, 10, or 20 mg/kg/b.w.) elevated cortical and hippocampal GABA and serotonin levels in a dose-dependent manner. In addition, piperine’s anticonvulsant effect suppressed the onset and duration of maximal electroshock-induced seizures in an epilepsy model [110]. Similarly, da Cruz et al. found that piperine treatment (2.5, 5, 10, or 25 mg/kg/b.w.) may modulate or activate GABA, glycine, and taurine neurotransmission and reduce serum and brain nitrite levels. Pilocarpine-induced reductions in anti-inflammatory and antioxidant activity and TNF-α levels have also been reported in an epileptic mouse model [111]. Similarly, Bukhari et al. reported that piperine treatment (30, 50, or 70 mg/kg/b.w.) had analgesic and anticonvulsant effects, possibly mediated via the opioid and GABAergic pathways in pentylenetetrazole- and picrotoxin-induced epileptic seizures models [135]. In the temporal-lobe epilepsy or post-status-epilepticus animal model, piperine treatment (25 mg/kg/b.w. for 10 days) restored serotonin levels and modulated the MAO and GABAergic pathways [112].

### 4.6 Black-pepper activity in stroke

Ischemic brain injury caused by stroke or cardiac arrest is a major cause of human neurological disability. Acute ischemia stroke results from thrombosis or embolism occluding a cerebral vessel to induce sudden loss of blood supply to a brain region [136]. Recent reports have extensively shown that dietary phytochemicals with beneficial health effects reduce the risk of stroke. Black pepper and its active metabolites have anti-ischemic activities and can treat cerebral ischemic injuries by modulating several signaling pathways.

Hua et al. observed that the dichloromethane fraction of black-pepper fruit extracts (100 or 200 mg/kg/b.w.) alleviated neurological deficits and increased protein levels of postsynaptic density protein 95 (PSD-95), phosphorylated calmodulin-dependent protein kinase II (p-CaMKII), calmodulin, and N-methyl D-aspartate receptor subtype 2B (NR2B) while preventing ischemia-induced cellular damage. Furthermore, immuno-histochemical analysis showed that black-pepper fruit extract significantly increased PSD-95 and synapsin-I (SYN-I) protein levels but decreased α-synuclein expression in the brains of permanent middle cerebral artery occlusion (pMCAO)-induced ischemic rats [113]. In addition, Zhang et al. reported that the dichloromethane fraction of black-pepper fruit extracts (50, 100, or 150 mg/kg/b.w.) reduced infarct volume, neurological score, and brain damage. Moreover, black-pepper fruit extracts downregulated ATG7 and p-AKT levels and upregulated p-mTOR levels in pMCAO-induced ischemic rats [114]. Furthermore, piperine supplementation (10 or 20 mg/kg/b.w.) had anti-ischemic activity, thereby reducing ischemia-induced cellular damage, neurological deficit, and cerebral infarction areas with less severe macro- and micro-cellular cerebral structural changes. In addition, piperine treatment inhibited BAX, caspase-3, caspase-9, and cytochrome-C release while enhancing Bcl-2 expression in pMCAO-induced ischemic rats [115]. Similarly, another study reported that piperine treatment (10 or 20 mg/kg/b.w.) reduced infarct volume and cortical neuronal loss and significantly decreased complement component 3, fibrinogen gamma chain, alpha-2-macroglobulin, and transferrin in the cerebral ischemia/reperfusion injury rat model [116].

Interestingly, Kaushik et al. reported that piperine treatment (10 mg/kg/b.w.) reduced mitochondrial dysfunction and had antiapoptotic potential by inhibiting BAX, cytochrome-c release, and caspase-3 activation, elevating antiapoptotic and Bcl-2 protein levels, and reducing IL-1β- and GFAP-positive cells in the cortex. It also improved behavioral performance and enhanced cell survival by restoring BDNF activity and its transcription protein, i.e., cyclic AMP response element binding protein (CREB), in ischemic stroke rats [117]. Similarly, Vaibhav et al. reported that piperine treatment (10 mg/kg/b.w.) significantly improved behavioral function; reduced the level of proinflammatory cytokines IL-1β, IL-6, and TNF-α; decreased COX-2 and NOS-2 expression; and reduced NF-κB activation in MCAO-induced ischemic rats [118]. Piperine dose-dependently increased cell viability and PI3K expression and significantly decreased p-PI3K, p-AKT, and p-mTOR levels in oxygen-glucose deprivation-induced HT22 cells. *In vivo* results showed that piperine treatment (10, 20, or 30 mg/kg/b.w.) had a neuroprotective effect on cerebral ischemia injury after 14 days. These findings indicate that piperine can inhibit the PI3K/AKT/mTOR pathway and autophagy, promote neurological recovery, and improve neuronal morphological structure in rats with ischemic stroke [119].

### 4.7 Black-pepper activity in depression and anxiety

Depression is a neuropsychiatric syndrome characterized by behavioral, psychological, and physiological symptoms with increased mortality and morbidity that also affects aging populations [137, 138]. Currently, available antidepressants are limited, and this has increased interest in alternative and complementary medicines. Indeed, several reports have shown that black pepper and its alkaloid piperine are potential antidepressants.

Black pepper and its bioactive compound piperine are commonly used as traditional remedies for depression and can prevent occurrence. Maneenet et al. reported that black-pepper fruit extracts combined with Kleeb Bua Daeng (100 or 500 mg/kg/b.w.) ameliorated behavioral impairments (e.g., hopeless behavior, sucrose intake test, tail suspension test [TST], and forced swimming test [FST]). It also promoted neurogenesis by upregulating BDNF and CREB expression in the frontal cortex and hippocampus. In addition, it significantly attenuated hypothalamic-pituitary-adrenal axis dysregulation by upregulating glucocorticoid receptor expression but downregulating serum- and glucocorticoid-inducible kinase 1 and FK506 binding protein 5 expression in unpredictable chronic mild stress (UCMS)-induced mice. Furthermore, black-pepper fruit extract with Kleeb Bua Daeng normalized proinflammatory cytokine expression, including TNF-α, IL-1β, and IL-6, and had an inhibitory effect on MAO A and B *in vitro* [120]. Similarly, Maneenet et al. showed that oral administration of black-pepper fruit extract with Kleeb Bua Daeng to UCMS-induced mice significantly improved their cognitive function. It also significantly decreased serum corticosterone and MDA levels but increased catalase and SOD activities in the frontal cortex and hippocampus [121]. Ghosh et al. subsequently reported that essential oil from the black-pepper fruit has antidepressant activity. Their behavioral studies using FST, TST, and the open-field test, along with neurochemical analysis, showed that black-pepper essential oil is a potent antidepressant at 5, 10, and 50 mg/kg/b.w. doses [139]. Similarly, methanolic black-pepper fruit extract decreased immobility in mouse TST and FST, with antidepressant effects observed at 100, 200, and 400 mg/kg/b.w. doses [72].

Mao et al. reported that piperine treatment (10 mg/kg/b.w.) upregulated mRNA and protein BDNF levels in the hippocampus and significantly increased sucrose consumption and decreased immobility time in the FST and TST in corticosterone-induced depression in mice [122]. Similarly, Mao et al. reported that piperine treatment (10 mg/kg/b.w.) significantly increased sucrose consumption and decreased immobility time in the FST. In addition, 5-HT and BDNF levels in the hippocampus and frontal cortex were significantly increased in UCMS-induced rats [123]. Rinwa et al. subsequently showed that piperine (20 mg/kg) with curcumin (100, 200, or 400 mg/kg/b.w.) significantly decreased immobility time in the FST and hyperactivity in the OFT of olfactory bulbectomized rats. In addition, it increased serum corticosterone levels and sucrose consumption, altered mitochondrial enzyme complexes, and decreased oxidative damage, proinflammatory cytokine TNF-α levels, and apoptotic factor caspase-3 levels but increased BDNF levels in the brain [140]. Black pepper also had a potential effect on anxiety in a mouse model. The administration of black pepper containing 30% β-caryophyllene (Viphyllin) at 50 or 100 mg/kg/b.w. doses improved brain antioxidant status, promoted neuronal cell survival, and inhibited NLRP3 inflammasome activation, thereby mitigating the dextran sodium sulfate-induced anxiety-like behavior of mice [141].

### 4.8 Black-pepper activity in neuroinflammation and neuronal cell death

Neuroinflammation is a defense mechanism associated with cell response in the CNS, including neurons, macroglia, and microglia [142]. Neuroinflammation characteristics are attributed to microglia activation, astrocytes in the brain parenchyma, the adaptive immune system, and inflammatory mediators such as cytokines, chemokines, and ROS/NOS [143]. Previous studies have suggested that a diet rich in natural products lowers the incidence of ANDs, in which inflammation is the core pathophysiological mechanism [144].

Bui et al. found that ethanolic black-pepper fruit extract had anti-inflammatory effects in a mouse model. The administration of ethanolic black-pepper fruit extract (200 mg/kg/b.w.) inhibited the expression of cytokines IL-4, IL-6, IL-1β, and TNF-α and increased IL-10 and interferon-γ secretion in ovalbumin-induced mouse homogenates [145]. Additionally, ethanolic black-pepper fruit extract prevented the activation of signal transducer and activator of transcription 3 and NF-κB/p65 signaling in the cytoplasm, leading to increased synthesis of anti-inflammatory T-helper (Th)1 cytokines and suppression of inflammatory Th2 and Th17 cytokines [146]. In another study, AD-induced rats treated with black pepper had significantly decreased brain and serum NF-κB levels [95]. Treating the dextran sodium sulfate-induced inflammatory bowel-disease mouse model with piperine (10, 25, or 50 mg/kg/b.w.) activated the pregnane X receptor, protecting against sodium sulfate-induced colitis by inhibiting the NF-κB-mediated proinflammatory response [147]. Similarly, Roshanbakhsh et al. found that piperine treatment (5, 10, or 20 mg/kg/b.w.) inhibited iNOS, TNF-α, IL1-β, and NF-κB expression and glial activation concomitant with significantly increased IL-10 expression [108]. Another study showed that levels of proinflammatory cytokines, including TNF-α, IL-6, IL-1β, and PGE2, were significantly attenuated by piperine in a time- and dose-dependent manner via inhibition of lipopolysaccharide (LPS)-induced NF-κB activation in BV-2 cells [148]. Treatment of an ischemia reperfusion-induced rat model with piperine (20 mg/kg/b.w.) significantly reduced plasma creatinine and urea-nitrogen levels, oxidative stress, renal histopathologic injuries, and proinflammatory cytokine expression by inhibiting the NF-κB pathway [149]. Wang et al. subsequently reported that piperine treatment (2.5, 5, or 10 mg/kg/b.w.) ameliorated D-galactose-induced neurochemical, neuroinflammatory, and cognitive alterations and reversed D-induced GSK-3β activation by modulating protein kinase C and PI3K/AKT signaling pathways in senescent mouse hippocampus [150].

## 5. Using nanotechnology to improve delivery and bioavailability of black pepper and piperine in age-related neurological disorders

**Figure 3.**
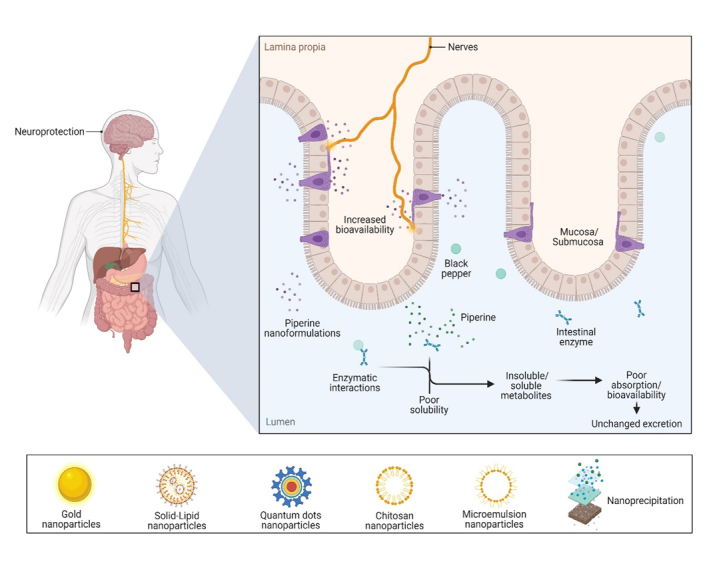
Black pepper and piperine nanoformulations pave the way for combating age-related neurological disorders, targeting different dysregulated mechanisms.

Drug delivery for CNS diseases is challenging, since the innate BBB and the blood-cerebrospinal fluid barrier impede drug delivery. Among new strategies for overcoming these limitations and successfully delivering drugs to the CNS, the nanotechnology-based drug-delivery platform offers a potential therapeutic approach to treat some common ANDs [151]. The nanoparticles (NPs) used as carriers are designed to deliver the phytochemicals to the target site with enhanced bio-efficacy and can cross the BBB more freely than larger particles [152]. NP drug-delivery systems can enhance neuronal AND management by diagnosing, monitoring, controlling, and repairing at the molecular level. NP treatments have many benefits, including suitable biodegradability and biocompatibility, improved therapeutic efficacy and pharmacokinetics, and decreased adverse drug effects. In addition, NPs can modulate major dysregulated pathways in ANDs, including oxidative stress, protein aggregation, neuroinflammation, and apoptosis [153]. Figure 3 illustrates the potential uses of black pepper and piperine nanoformulations against different ANDs.

Etman et al. reported that piperine-loaded microemulsion nanocarriers (PIP-ME) at a dose of 2.5 mg/kg/b.w. protected against colchicine-induced memory dysfunction in AD mice. Here, oral PIP-ME administration increased drug efficacy, solubility, and stability and enhanced drug delivery to the brain. Additionally, PIP-ME treatment enhanced memory performance, decreased AChE activity, reduced oxidative stress, and inhibited neuroinflammation and apoptosis induced by caspase-3, suggesting that PIP-ME has a better therapeutic outcome than free piperine [154]. Similarly, Elnaggar et al. reported that Tween-modified monoolein cubosome (T-cub)-loaded piperine NPs at a dose of 2.5 mg/kg/b.w. had potential therapeutic roles in AD. Treatment with these oral T-cub piperine nanoformulations greatly increased oral absorption, bioavailability, and enhanced brain targeting efficacy over free piperine. In addition, T-cub piperine nanoformulations prevented cognitive dysfunction and enhanced memory function; they also showed potential oxidative damage suppression and significantly decreased AChE, anti-inflammatory, and antiapoptotic activity, indicating the cessation of AD progression [155]. A similar study also reported that intranasal piperine-loaded chitosan NPs (CS-NPs) significantly alleviated piperine nasal irritation, reduced oxidative damage, inhibited AChE activity, significantly improved cognitive functions, and showed antiapoptotic and anti-inflammatory effects in colchicine-induced AD rats. They also reported that piperine-loaded CS-NPs did not show any toxicity in the brain [156].

Yusuf et al. investigated polysorbate 80-coated piperine solid-lipid NPs (PS-80-PIP-SLN) with an emulsification-solvent diffusion method as a brain-targeted drug for treating AD. Treatment with PS-80-PIP-SLN at a dose of 2 mg/kg/b.w. provided effective delivery, crossing the BBB and reducing the amyloidal content and tangles in the brain. These observations were confirmed by histopathology and may have helped increase ACh levels by preventing Aβ accumulation [157]. Chonpathompikunlert et al. reported that nitroxide radical-containing NPs coupled with piperine enhanced cell viability, reduced ROS levels, and augmented antioxidant enzyme activity, thereby preventing apoptosis in Aβ-induced SH-SY5Y cells [158].

Piperine-coated gold NPs (AuNPs) suppressed oxidative stress and mitochondrial dysfunction, thereby inhibiting apoptotic cell death in paraquat-induced SH-SY5Y cells and a *Drosophila melanogaster* PD model. Lower concentrations of piperine-coated AuNPs (10, 50, or 100 μM) effectively reversed paraquat’s lethal effects; they provided significant cytoprotective effects by suppressing oxidative stress and mitochondrial dysfunction while maintaining MMP, thereby inhibiting apoptotic cell death in paraquat-induced SH-SY5Y cell models. In addition, piperine-coated AuNPs improved locomotor function and life span; reduced oxidative stress, JNK and caspase-3 activity, and p53 levels; and increased parkin expression in a *D. melanogaster* PD model. These results highlight the protective function of piperine-coated AuNPs in paraquat-induced SH-SY5Y cells and a *D. melanogaster* PD model [159].

Anissian et al. reported that piperine-loaded chitosan-sodium tripolyphosphate (CS-STPP) NPs showed potential therapeutic activity in the pentylenetetrazole (PTZ)-induced kindling epilepsy model. The encapsulation of piperine-coated CS-STPP NPs led to improved water solubility and bioavailability. Furthermore, lower concentrations (5 or 10 mg/kg/b.w.) modulated behavioral performance, significantly increased NeuN-expressing cells, and reduced neuronal cell death and activated astrocytes in the dentate region of the hippocampus compared to the free piperine in the pentylenetetrazol-induced kindling epilepsy animal model [160]. Another study compared free piperine with nanosized piperine prepared by the nanoprecipitation method, finding that the latter had a much better dissolution rate, improving its oral bioavailability and brain delivery. In addition, piperine NPs modulated total move distance and larval locomotor activity, suggesting that they have promising anti-epileptic effects in PTZ-induced epileptic zebrafish and mouse models [161]. A similar study encapsulated piperine in copper-oxide quantum dots coated with hyaluronic acid (HA)/poly(lactic-*co*-glycolic acid; PLGA) *in vitro* and *in vivo* and investigated its neuroprotective and anticonvulsive efficiency in the PTZ-induced kindling model. Their results showed that piperine-loaded CuQDs@HA/PLGA nanocarriers had an anticonvulsant activity due to their greater water solubility and increased oral bioavailability. Additionally, piperine-loaded CuQDs@HA/PLGA nanocarriers showed increased viability and zero cytotoxicity in brain microvascular endothelial cells (at the doses examined) and had a better ability to enter cells than free piperine. Moreover, piperine-loaded CuQDs@HA/PLGA nanocarriers suppressed myoclonic jerk latency, generalized clonic-tonic seizures, and seizure behavior better than free piperine in the PTZ-induced kindling model [162].

## 6. Clinical study of black pepper and piperine activity in age-related neurological disorders

Black pepper and its active constituents have been clinically studied for different diseases. Among the different neurological disorders, clinical studies have investigated the effects of black pepper and its bioactive constituent piperine in neuropathic pain, depressive disorders, cognition, memory, and stroke.

A one-month randomized control study conducted between June 2001 and March 2002 included 109 eligible patients with physical handicaps mainly due to cerebrovascular disease. Nasal inhalation of black-pepper essential oil, which can activate the insular or orbitofrontal cortex, improved the reflexive swallowing movement. Therefore, it might beneﬁt older poststroke patients with dysphagia regardless of their consciousness/physical levels and mental status [163]. An eight-week open randomized control trial on 141 patients with neuropathic pain showed that consuming a multi-ingredient formula (Lipicur), consisting of 400 mg of lipoic acid, 400 mg of curcumin phytosome, and 4 mg of piperine, reduced neuropathic pain by >66% in patients with lumbar sciatica or carpal tunnel syndrome [164]. Similarly, a randomized double-blind placebo-controlled study examined the effect of piperine combined with resveratrol on cerebral blood flow (CBF) parameters and cognitive performance in 23 healthy adults (four males and 19 females). Their results showed that, when co-supplemented, piperine (20 mg) and resveratrol (250 mg) significantly improved CBF during task performance compared to placebo and resveratrol alone. However, cognitive function, mood, and blood pressure were unaffected [165]. A six-week open-label trial on patients at high risk of major depressive disorder showed that consuming curcuminoids containing piperine led to significantly greater reductions in total hospital anxiety and depression scale scores, anxiety and depression subscale scores, Beck depression inventory-II total score, and somatic and cognitive subscale scores [166]. A meta-analysis of eight clinical trials conducted on 626 patients between four and eight weeks indicated that curcuminoids can significantly reduce plasma MDA levels and increase serum SOD activity in red blood cells without affecting glutathione peroxidase (GPx) activity. Moreover, curcuminoids (600 mg/d) combined with piperine (10 or 15 mg/d) showed better activity than curcuminoids alone [167].

## 7. Toxicological insights of black pepper and piperine: Preclinical studies

Natural food products have been considered safe for human health for centuries. In Asian countries, many foods prepared at home or purchased from commercial bakeries contain spices, including black pepper, to improve their taste. Therefore, daily consumption of black pepper in different foods may not produce any toxic effects, though it may be an “irritant” if consumed in higher amounts.

It was recently reported that oral administration of aqueous black-pepper fruit extract (5,000 mg/kg/b.w.) to male and female Sprague-Dawley rats did not produce signs of toxicity, mortality, behavioral changes, or histopathological changes in internal organs. The sub-chronic toxicity of black pepper has also been assessed in rats. Oral administration of aqueous black-pepper fruit extract (300, 600, and 1,200 mg/kg/b.w.) continuously for 90 days led to no abnormalities in the test groups compared to the control group [168]. Mahdy et al. gave black-pepper seeds at high (750 mg/kg/b.w.), medium (375 mg/kg/b.w.), and low (187.5 mg/kg/b.w.) doses to male Sprague-Dawley rats, finding that they did not cause death within three months [169]. Furthermore, a recent study evaluated the acute oral toxicity of methanolic black-pepper extracts and their potential health risks on various rodent organs (eyes, nose, ears, and fur). Their results showed that oral administration of methanolic black-pepper extracts (1,000, 2,000, or 3,000 mg/kg/b.w.) had no significant morphological effects on rodent eyes, ears, nose, and fur. In addition, methanolic black-pepper extracts were stable at a single dose of up to 3,000 mg/kg/b.w., indicating that black pepper is safe for oral administration in rodents [72]. Similarly, the polyherbal mixture of black-pepper seeds (500, 1,000, or 1,500 mg/kg/b.w.) was investigated in streptozotocin-induced diabetic rats. Their acute toxicity experiment showed that administering the polyherbal black-pepper seed mixture for 14 days did not cause any abnormalities in animals. In addition, their sub-chronic toxicity experiment showed that administering polyherbal black-pepper seed mixture (500, 1,000, and 1,500 mg/kg/b.w.) for 28 days did not alter the monitored biochemical, histological, and hematological parameters in the kidney and liver of diabetic rats [170].

Rao et al. observed that piperine is non-toxic up to 100 mg/kg/b.w., but they observed that the amount that killed 50% of the test sample (LD_50_) for a single intravenous administration was 15.1 mg/kg/b.w. for adult mice. However, piperine oral administration at doses of 330 and 514 mg/kg/b.w. have been reported as the LD_50_ values for rats and mice, respectively. In contrast, while piperine administration decreased serum protein levels, it increased aspartate aminotransferase and alkaline phosphatase levels (i.e., signs of significant liver damage) in albino rats [171]. Recently, genotoxic *in vivo* and *in vitro* studies have found that piperine at doses of 143.5, 287, and 574 mg/kg/b.w. for two days have no adverse effects on erythropoietin levels, core body temperature, hematological indicators, or organ weights, concluding that piperine is not genotoxic [172]. Reproductive toxicity studies showed that piperine (at doses up to 75 mg/kg/b.w.) does not affect the reproductive functions of male germ cells, spermatogenesis, and epididymis enzymes. However, higher piperine doses significantly reduces sialic levels, epididymis enzymatic functions, and testis development in pubertal rats [173, 174, 175]. In contrast, Malini et al. found that oral piperine administration (5 or 10 mg/kg/b.w.) for 30 days in rats causes severe germ-cell degeneration, while piperine at 10 mg/kg/b.w. severely affects spermatids and spermatocytes and causes structural changes in the seminiferous tubular, lowering concentrations of caput and cauda epididymis sperm in rats [176]. An immunotoxicity study orally administering piperine (1.12, 2.25, and 4.5 mg/kg/b.w.) for five days found no obvious toxic effects in Swiss male mice, suggesting that piperine is immunologically safe at 1.12 mg/kg/b.w. However, higher piperine doses induced adverse effects, such as decreased serum primary antibodies and antibody-forming cells, and a suppressed mitogenic response in B-lymphocytes [177].

## 8. Conclusion and future perspectives

**Figure 4.**
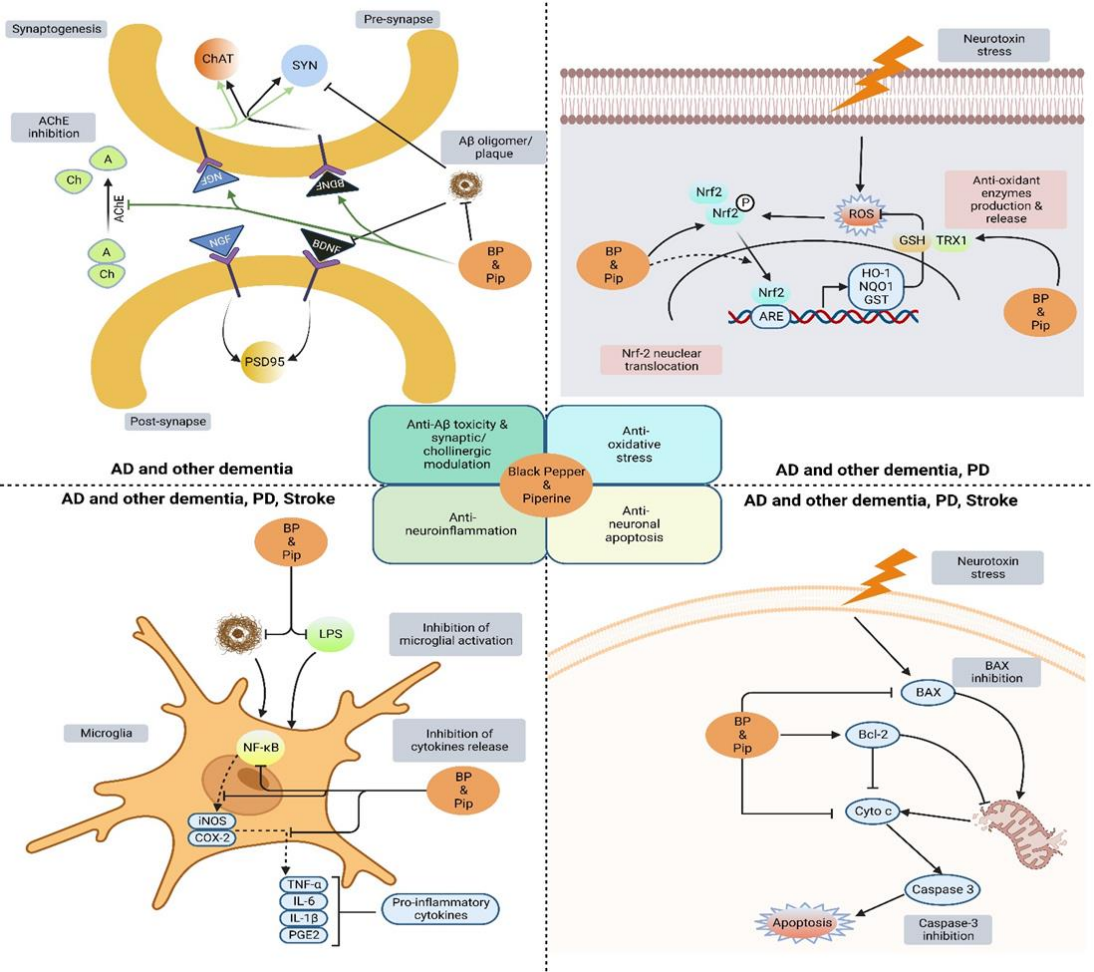
**Neuroprotective potential of black pepper and its bioactive compound piperine in age-related neurological disorders.** Black pepper and piperine show various neuropharmacological effects via antioxidative stress, anti-neuroinflammation, cholinergic function, anti-Aβ toxicity, anti-neuronal apoptosis, and synaptic function modulation. The block lines () and arrows (→) denote inhibition and stimulation actions by black pepper and piperine, respectively. Key: Aβ, amyloid beta; HO-1, heme oxygenase; Nrf2, nuclear factor erythroid 2-related factor 2; NF-κB, nuclear factor kappa B; ROS, reactive oxygen species; iNOS, inducible nitric oxide synthase; COX-2, cyclooxygenase-2; LPS, lipopolysaccharide; AChE, acetylcholinesterase; IL-1β, interleukin-1β; TNF-α, tumor necrosis factor-α; IL-6, interleukin-6; PSD-95, postsynaptic density protein 95; GSH, glutathione; GPx, glutathione peroxidase; Bcl2, B-cell lymphoma 2.

In this review, we summarized the recent evidence for the pharmacological potential of black pepper and its active constituent, i.e., piperine, in the treatment of ANDs. Several clinical drugs improved brain health in this population but also suggested undesirable side effects. In exploring natural alternatives to improve the quality of life of older adults, black pepper is an excellent plant-derived food material for this purpose. Black pepper has been examined for its therapeutic efficacy in AND models and NDD-related pathological conditions. It ameliorates disease-specific symptoms and pathological changes by controlling the AND pathogenesis. Furthermore, the black pepper-derived active compound piperine showed various anti-AND effects in preclinical studies, demonstrating its neuropharmacological actions along with their modes of action. Figure 4 summarizes the neuroprotective potential of black pepper and its bioactive compound piperine in ANDs.

This review has reported that black pepper and its active ingredient, i.e., piperine, can potentially increase the bioavailability of some phytochemicals with low toxicity. However, piperine has some major issues limiting its clinical use, including low water solubility and poor dissolution. Its hydrophobic nature and poor aqueous solubility are the primary reasons for its poor bioavailability. This problem is a major hurdle for its translation from lab to clinic, since its low aqueous solubility poses a rate-limiting step in its absorption process. Therefore, new formulations are required to enhance piperine’s aqueous solubility and make it more bioavailable. To date, very little has been done in this regard, and studies dealing with piperine formulations for medicinal applications are rare. Nevertheless, alternative delivery systems have been developed to address this problem.

Drug-delivery systems using nanoformulations and encapsulations as carriers have successfully encapsulated black pepper and piperine to treat ANDs such as AD, PD, and epilepsy. This review discussed the different NP types, including AuNPs, solid lipid, quantum dots, chitosan, and microemulsion NPs. We summarized studies that have used these NPs as carriers for black pepper and piperine administration to enhance their aqueous solubility, stability, bioavailability, target specificity, and bioactivities. Therefore, novel pharmaceutical delivery strategies may overcome piperine’s solubility and bioavailability issues, enhancing its potential effectiveness in humans. Further preclinical studies are needed on NPs for piperine delivery in other ANDs (i.e., HD, ischemia stroke, and MS) to evaluate their efficacy, solubility, and bioavailability and address potential safety concerns.

Another important factor to consider is the dose dependency of piperine’s pharmacological effects. Although lower doses of piperine (<20 mg/kg/b.w.) show a wide range of therapeutic activities, such as anti-AD, anti-PD, and antidepressant effects, higher doses (≥20 mg/kg/b.w.) show adverse effects on the body [75, 178, 179]. Therefore, its bio-absorption, metabolism, and toxic side effects may vary based on the delivery medium. Consequently, animal studies are needed to determine safe piperine doses for ANDs. In addition, studies on consumption patterns are still needed to establish the basis for the post-consumption effects of piperine ingested through human diets.

In conclusion, ANDs remain challenging to treat because their etiology remains largely unknown. Moreover, their pathogenesis represents a complex network involving multiple signaling pathways but no feedback system to adjust imbalances after disease onset. Numerous studies are being conducted on the effects of natural products on brain disease pathologies, such as protein misfolding, oxidative stress, mitochondria dysfunction, and inflammation. Notably, black pepper and its active constituent piperine ameliorated multiple pathological brain-degeneration conditions in various *in vitro* and *in vivo* models through their antioxidant, anti-inflammatory, and protein-modifying properties. Black pepper, as a multi-targeting agent, may be a promising therapeutic candidate for treating ANDs and brain aging. Additionally, its main constituents, including piperlonguminine, piperanine, pipercallosine, dehydropipernonaline, pipernonatine, retrofractamide B, pellitorine, guineensine, chabamide, retrofractamide A, isopiperolein B, 6,7-dehydrobrachyamide B, cepharadione A, paprazine, piperolactam D, sylvamide, chavicine, 10-tricosanone, pellitorine, piperettine, piperettyline, feruperine, and piperine analogs such as HJ105, HJ22, and 3B have also been shown to contribute to its pharmacological efficacy. Therefore, the standardization of black-pepper samples and their constituents by high-performance liquid chromatography and gas chromatography-mass spectrometry will provide invaluable insights into using black pepper as a potent natural medicine.
